# A Low-Cost Active Reflector for Interferometric Monitoring Based on Sentinel-1 SAR Images

**DOI:** 10.3390/s21062008

**Published:** 2021-03-12

**Authors:** Guido Luzi, Pedro F. Espín-López, Fermín Mira Pérez, Oriol Monserrat, Michele Crosetto

**Affiliations:** Centre Técnologic de Telecommunicacions de Catalunya (CTTC/CERCA), Division of Geomatics, Av. Gauss 7, Castelldefels, E-08860 Barcelona, Spain; pedro.espin@cttc.es (P.F.E.-L.); fermin.mira@cttc.es (F.M.P.); oriol.monserrat@cttc.es (O.M.); michele.crosetto@cttc.es (M.C.)

**Keywords:** SAR, interferometry, backscattering, radar reflectors, deformation

## Abstract

The effectiveness of radar interferometric techniques in non-urban areas can often be compromised due to the lack of stable natural targets. This drawback can be partially compensated through the installation of reference targets, characterized by a bright and stable radar response. The installation of passive corner reflectors (PCR) often represents a valid aid, but these objects are usually cumbersome, and suffer from severe weather conditions; furthermore, the installation of a PCR can be difficult and costly, especially in places with hard accessibility. Active reflectors (AR) represent a less cumbersome alternative to PCRs, while still providing a stable phase response. This paper describes the design, implementation, and test of an AR prototype, designed to operate with the Sentinel-1 synthetic aperture radar (SAR), aimed at providing a fair performance/cost benefit. These characteristics, obtained through a tradeoff between the use of off-the-shelf components and a simple architecture, can make the setup of a dense network (i.e., tens of devices) in the monitored areas feasible. The paper reports the design, implementation, and the analysis of different tests carried out in a laboratory, and in a real condition in the field, to illustrate AR reliability and estimate its phase stability.

## 1. Introduction

The use of Sentinel-1 SAR images for interferometric synthetic aperture radar (InSAR) in non-urban areas is often challenging because most of these areas are covered by vegetation or heterogenous surfaces, which are geometrically and dielectrically instable, and hence have a low coherence. The deployment of stable artificial targets in these areas can remarkably improve the situation. Strong reflectors, with a stable amplitude and phase response, can be classified as persistent scatterers [1,2]; thus, provide a reference for interferometric applications. The presence of such targets in the area allows a better image interpretation and supports the calibration process. Monitoring areas with a high density of buildings and infrastructure can benefit from the presence of a huge of these reflectors. On the other hand, this is not happening when the monitored areas are mountain regions, rural areas, glaciers, or snow-covered areas. In these cases, the installation of artificial reflectors, usually passive corner reflectors (PCR), can be crucial [3]. A PCR is an object with a simple geometrical shape, designed to provide, when properly installed, high radar reflectivity and a stable phase over time. PCRs are usually assembled with metal plates, with a large size, with respect to wavelength. The shape and orientation of their surfaces maximize the energy reflected towards the radar. Different types of PCRs are used, the most common are triangular trihedral (TT) corner reflectors (CR) [4,5,6]. Innovative shapes and concepts have been recently proposed [7,8], but they demand laborious installation and, sometimes, specific processing procedures. The main drawback of a PCR is its bulky size and weight, in addition, its response can suffer from adverse weather conditions, such as heavy rainfall, snowfall, or strong winds. The use of PCRs integrated with other sensors, such as Global Positioning Systems (GPS)have also been recently reported in the literature [9,10,11]. Finally, innovative PCRs, based on the use of specifically etched dielectric surfaces instead of metal plates, have also been proposed for ground-based SAR [12], but to the authors’ knowledge, they have not been extended to satellite applications. 

The installation of a PCR can be difficult when accessibility to the installation is hard, and weather conditions can jeopardize their performance. An alternative to PCR is represented by the installation of active reflectors (ARs). These systems have been introduced since the 1990s for SAR system calibration [13,14,15,16], and the interest was renewed when data from spaceborne radar for Earth Observations became available to researchers and users [17,18]. Nevertheless, only in the last decade did the idea to provide commercial ARs to use in spaceborne SAR applications spread [19,20,21], and the application extended for spaceborne SAR, working at different bands [22]. With respect to a PCR, an AR is smaller and lighter, but its reliability is often reduced by the need of a power source, usually a battery buffered by solar panels, and its phase stability demands careful design. Innovative concepts are currently under investigation based on the use of reflect arrays [23], but to the authors’ knowledge, until today, no operative applications to spaceborne radar interferometry have been proposed.

This study does not take into consideration complex transponders developed to assist—during and after the commissioning phase—the calibration and test of space sensors, representing, in terms of cost, performance, and use, very different systems. Nowadays ARs designed for field installations are available from the market at a moderate cost. These systems are compact enough, and they provide users an advanced interface to allow the control of some acquisition parameters. An outstanding example is the MetaSensing Electronic Corner Reflector at C-band (ECR-C) [24]. The performance, cost, and operating capability of the ARs discussed here differ from them. The goal is to make available a sensor alternative to these systems because their price is still too high to allow for dense coverage of wide areas in an operational context. The low consumption and basic requirement of the AR proposed here aims to provide a long-term autonomy, especially when buffering power sources, as solar panels may not be enough to assure a long and continuous operation. It is worth noting that this issue is particularly critical when ARs are installed in certain areas, such as glaciers or snow covered areas, where reaching the installation site is costly, not easy, and unsafe. Considering the state-of-the-art of the ARs’ development, this study proposes to stimulate a novel approach: the AR achieves a high linearity via a low-cost design. The performance of the device is not optimum, but a wide dissemination in large areas is feasible, especially in low coherence areas, where it is difficult to install and power the device. The goal is to provide many reference points for deformation monitoring through SAR interferometry. In this paper, we verify the feasibility of this approach with an accurate description of a study, which includes the design, the implementation of an AR, and the analysis of some experimental tests, including a real case. 

The AR was designed with a simple rationale, which basically consists of two compact microstrip antennas (size 14 cm × 14 cm), and a radio frequency (RF) section to provide the requested signal gain. One of the design requirements is to maintain the current absorption very low, to allow the use of a power system with a small capacity 12 V battery (7 Ah) and a small (25 cm × 24 cm) photovoltaic panel (5 W and 25 × 24 cm size), and provide long autonomy in case of a lack of buffering power. The size of the case of the prototype here discussed is 52 cm × 32 cm × 7 cm. Phase stability has been achieved with a tradeoff among low-cost, simple functioning, and easy and rugged hardware, using off-the-shelf components. This choice allows for minimizing the cost of most of the components. In addition, the antennas, designed and developed at Centre Tecnològic de Telecomunicacions de Catalunya (CTTC), have also an implemented a cost, lower than 10% of the cost of the entire system. A rough estimate of the total cost of the current version of the AR discussed here is less than 1000 euros. These goals have been achieved with a simple radio frequency (RF) architecture, based on linearity and neatness, and taking care of all the aspects of the overall design. In order to evaluate its performance, the implemented device was tested at different levels and conditions. First, the system without antennas has been tested in a laboratory to check the performances of the RF amplifying section. Then, the performance of the entire apparatus was measured through a field experiment carried out in a controlled environment, with temperature variations between 10 °C and 35 °C, at short distances, and using a signal, simulating that of Sentinel-1. Finally, the AR was installed in an agricultural site, in real conditions, processing almost a one-year long slot of Sentinel-1 images, i.e., more than 50 Single Look Complex (SLC) images, in interferometric wide (IW) mode, descending orbit geometry. The stability of the AR amplitude and phase response has been analyzed, paying attention to the influence of the air temperature.

This paper is organized in five sections: this introduction, followed by a brief recall of the working principle of an AR, including a description of some design details and of the applied methodology, as described in Section 2. Section 3 contains the description of the basic tests carried out in laboratory and in a controlled environment, while in Section 4 we report on the processing and the results obtained in real conditions, based on Sentinel-1 images processing. The discussion about these results and the performance of the AR in each case are presented in Section 5. A short conclusion finalizes the paper.

## 2. Materials and Methods

In this section, a short description of the working principle is provided. Then, a design rationale is discussed. Details about the antennas and the amplifying section—the most critical points of the device—are also commented on. The methodology used to test the AR is also described.

### 2.1. The Working Principle

**Figure 1:**
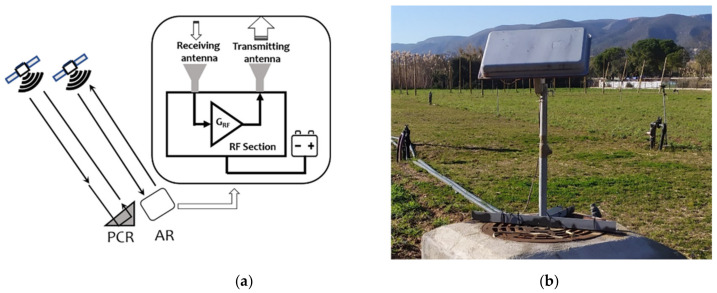
Working principle of the active reflectors (AR) (**a**). Picture of the AR installed in the field (**b**).

The main goal of this study is to report the feasibility of a low-cost, reliable and easy to use AR, able to assist InSAR use of Sentinel-1 SAR images. The simplest AR scheme consists of a receiving antenna, an amplifying section, and a second antenna. The signal received from the satellite is re-transmitted after an amplification towards the same satellite. The essential working principle is shown in Figure 1, which includes a picture of one of the tested ARs. This AR was specifically designed for the Sentinel-1 SAR sensor, operating at C-band (5.405 GHz ± 50 MHz). However, it can also work for Radarsat2 and other C-band sensors whose bandwidths are not drastically different from that of Sentinel-1. The AR can work with a single polarization combination selected among the linear co- and cross-polarizations: vertical/vertical (VV), vertical/horizontal (VH), horizontal/vertical (HV), or horizontal/horizontal (HH). As for passive targets, to estimate the strength of the signal received by the satellite, an equivalent radar cross section (RCS) is defined [25]. To detect a target and accurately analyze its response, its RCS must be higher than a threshold related to the radar brightness of the background. This first requirement was chosen on the basis of data available from the literature [4]. In land surface monitoring, the radar response of the background is highly variable. For this reason, we decided to take, as reference, the RCS of PCRs successfully installed in previous SAR monitoring surveys. We took, as a starting requirement for the AR design, the RCS value reported in [4] for Sentinel-1 (Table 1): 40.4 dBm^2^. It is worth noting that it corresponds to the RCS of a TT CR, whose size is 1.7 m. 

### 2.2. The Design

The radar cross section of an AR can be determined by characterizing the gains of its components. We used the relationship obtained from the radar equation used in [25]. According to the simple scheme depicted in Figure 1, the equivalent RCS of the AR, which depends on the gain of the two antennas, the gain of the amplifying section, and on the wavelength of the radar signal, can be obtained using the following simple formula:

(1) $$RCS=G_{\mathrm{RF}}\;\frac{G_{\mathrm{tx}}\;G_{\mathrm{rx}}}{4\pi }\lambda^{2}$$

where: *G*_RF_: gain of the RF amplifying section. *G*_tx_, *G*_rx_: gain of the transmitting and receiving antennas, respectively. λ: wavelength of the received signal.

We used two identical antennas, so: *G*_tx_ = *G*_rx_. Considering that the RCS of the AR must be > 40 dBm^2^, and that the measured gain of the patch array antenna specifically designed and implemented for the AR is greater than 17 dB (see Section 2.3.1), we can calculate through Equation (1) the required *G*_RF_. This value must not exceed the coupling between the two antennas, to avoid an auto feed of the AR due to leakage between them. The antenna separation was set to 30 cm in order to reduce the encumbrance of the device. With this geometry, the coupling calculated through simulation, and measured in an anechoic chamber, is lower than −50 dB; thus, permitting a maximum *G*_RF_ of 50 dB. The gain value set for the AR design is 42 dB, a value that we consider largely conservative.

### 2.3. The Implementation

#### 2.3.1. The Antenna

**Figure 2:**
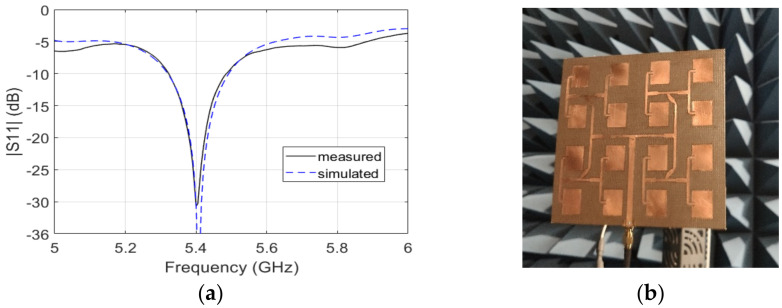
Picture of the C-band patch array designed and implemented for the AR (**a**). S_11_ of the antenna: solid and dashed lines show the measured and simulated data, respectively (**b**).

Observing Equation (1), it is clear that the gain of the antennas is of major concern, because fixing the maximum RF gain, the higher the gain, the lower the *G*_RF_. The power consumption of the device increases as the *G*_RF_ increases, so by reducing *G*_RF_, we also reduce the current absorption, one of the goals of this design. Furthermore, we can reduce the number of amplifying stages, minimizing the potential instability related to the matching between the different RF components, a factor that can worsen the phase stability. A specific patch array antenna was developed for this system. The size of a single antenna, whose picture is shown in Figure 2, is 14 cm × 14 cm. It consists of a 4 × 4 linear patch array, designed to provide a gain not lower than 17 dB in the operating bandwidth of the AR. 

**Figure 3:**
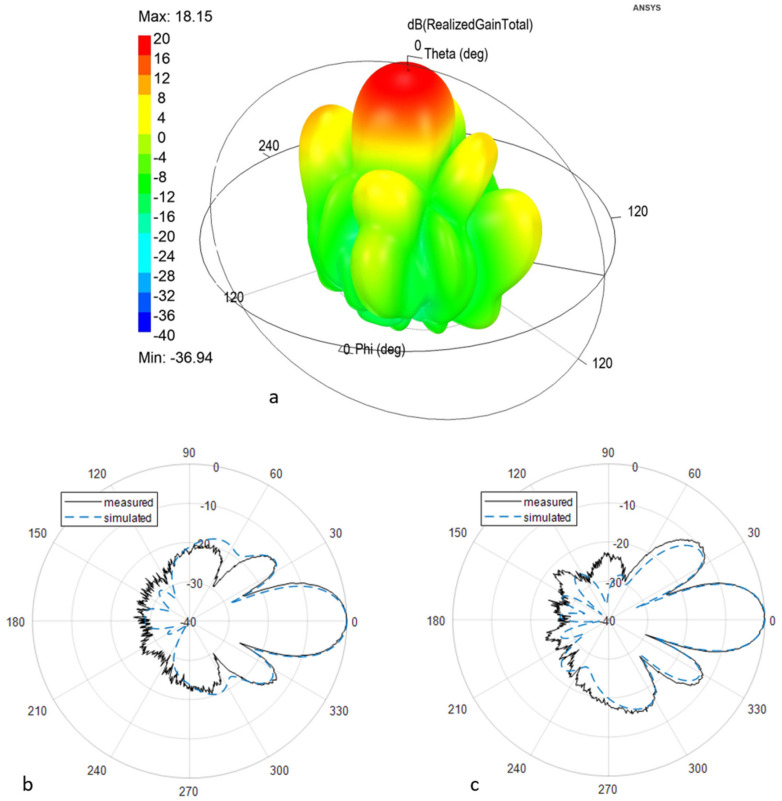
Antenna radiation pattern at 5.405 GHz. (**a**) Simulated three-dimensional (3D) surface; (**b**) polar diagram: simulated (dashed) and measured (solid), H plane; (**c**) polar diagram: simulated (dashed) and measured (solid), E plane.

A good standing wave ratio (SWR) is mandatory to reduce losses and reflections, and hence, noise, which can make the system more unstable and sensitive to temperature variations. S_11_, the parameter conventionally used to describe the matching performance of the antenna, was measured and simulated at the antenna connector. The measured value below −20 dB in the operating band assures good matching between the two antennas and the amplifying section; the measured S_11_ is compared to the simulated behavior. The antenna patterns, shown in Figure 3 in different representations, were measured in an anechoic chamber using a calibrated standard horn: the maximum gain is 17.3 dB. The value expected from the simulation, 18 dB, is very close; thus, demonstrating a good design and manufacturing [26].

#### 2.3.2. The Amplifying Section

**Figure 4:**
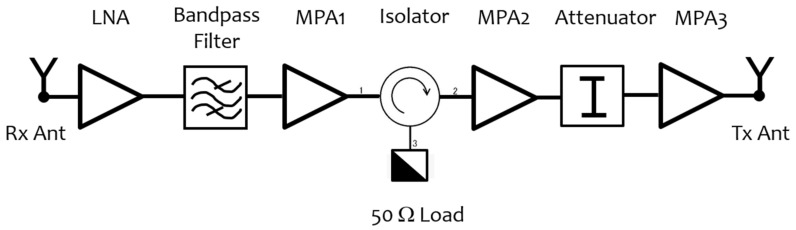
Diagram of the radio frequency (RF) section.

According to the RCS requirement, the antenna gain, and Equation (1), the required gain for the amplifying section must be greater than 40 dB and not exceed 50 dB. This value was obtained by designing this part of the system using components selected according to the requirements of a low power consumption. A band-pass filter is also necessary to avoid interferences from external sources, such as wireless or mobile networks. Different prototypes of the RF section were implemented and characterized in order to test different amplifiers and design schemes. The final architecture used off-the-shelf components with a negligible cost of the single RF components. The RF scheme is composed of a low noise amplifier (LNA) just after the receiving antenna, followed by a band-pass filter (5150–5990 MHz), and three amplifiers here indicated as Medium Power Amplifier (MPA), also with a low noise figure. The nominal gain of the amplifiers is about 15 dB each. Laboratory tests suggested adding an attenuator and an isolator to reduce the risk of saturation and optimize the matching among the different components. The implemented prototype is based on the use of evaluation boards of commercial low-cost components, but it is planned that the final version will be integrated in a single board. In Figure 4, a block diagram of the AR is shown. The overall system’s current absorption at 12 V is very low: < 45 mA; this allows keeping the device always switched on, operating as much as possible in a thermal and electronic steady condition, limiting the amplitude and phase fluctuations. This operating mode differs from that used in the MetaSensing ERC-C model [24] and other systems [20], which periodically switch on/off the RF section. In these systems, according to the satellite scheduling, the AR is switched on only periodically before the satellite passage and switched off until the next satellite`s passage. To make a quantitative comparison, the AR described here consumes the same energy of the ECR-C when it is in idle condition, as derived from [24]. To improve the power autonomy and reduce the consumption, the design does not include any further functionalities of the system different from the signal amplification. To reduce the power demand, the device does not include microprocessors, sensors, such as GPS/GNSS, or internal storage units. The only sensor present in the system is an autonomous temperature sensor whose data can be downloaded from a USB port in the external panel of the AR, and used to correct the sensor in case of unacceptable phase drift due to temperature variations, as will be explained in the following sections. 

The external size of the device used for this study, which is shown in Figure 1, is 52 cm × 32 cm × 7 cm, and it is mounted on a rotating arm. An advanced, more compact, version of the case containing the system has been recently developed and it is under test; it includes internally to the case, the frame to select the elevation angle, avoiding the need of an external rotating arm in the installation site.

### 2.4. Methodology

To test the performance of the AR, three main steps were taken. First, an indoor laboratory experiment was carried out to analyze the response, amplitude, and phase of the RF section, without the antennas, at different temperatures. Then, a test of the entire sensor was carried out, arranging a setup to evaluate the thermal stability of the AR at a short range, and using as input a frequency modulated continuous wave (FMCW) signal generated by a vector network analyzer (VNA). Finally, the prototype was installed in real conditions, together with a PCR. The AR and the PCR were installed in an experimental agricultural field of the campus above a concrete base (see Figure 1). A first check of the visibility of the AR in the Sentinel-1 image 1 was carried out using the Sentinel Hub browser provided by Synergy for European space Agency (ESA) [27]. The signal to clutter ratio (SCR) was then calculated to evaluate the potential accuracy on phase retrieval in the experimental conditions using a method similar to that proposed by other authors [3,4], using an amplitude Sentinel-1 image. Based on the use of the measured and calculated RCS of the PCR, the RCS of the AR was estimated, comparing the amplitude response of the two targets. Finally, using a consolidated in-home processing chain [28] developed to retrieve the deformation of land surfaces through differential SAR interferometry, the variation of the phase of the AR versus the acquisition dates was analyzed. The interferometric processing applied to retrieve the differential phase uses, as reference, the phase response of some pixels corresponding to stable buildings, located a tens of meters away from the AR to reduce the atmospheric effect (as low as possible). To estimate the AR phase stability, the cumulated displacement was calculated. A set of 55 Sentinel-1 images, SLC IW mode, were used (data downloaded from Copernicus hub [29]). Meteorological data provided by the meteorological service of the Catalonia Government [30] were used to study the phase versus temperature behavior.

## 3. Experimental Test of the Prototype

In this section, different tests carried out to evaluate the performance of the AR are reported. The first one consists of an indoor laboratory experiment aimed at a basic check of the characteristics of the amplifying section, the core of the device. A controlled environment experiment is then reported to detail the behavior of the entire AR, when the internal temperature of the case was changing. 

### 3.1. The Laboratory Test

#### 3.1.1. The Setup

The first step to evaluate the performances of the AR consists of measurements of its gain (*G*_RF_), using a vector network analyzer (VNA) (Agilent E8257D) to generate an FMCW signal. The measuring configuration uses a minimum frequency f_min_ = 5.2 GHz, and a maximum, f_Max_ = 5.6 GHz, with 201 frequency points. This swept bandwidth includes the RF bands used by most of the C-band spaceborne SAR systems. Different values of input power were used to test the linearity of the device (input power range: −80 dBm to −20 dBm), widely including the signal intensity expected for the actual Sentinel-1 input signal. The *G*_RF_ measured @ 5.4 GHz is 42dB, a value compliant with the design requirements. Once the conditions required to provide an adequate RCS of the AR were verified, the phase stability was first checked, at a stable temperature, using a mixer as a phase detector in a standard homodyne configuration, which provided an IF signal proportional to the phase difference between the input and output of the AR.

The achievement of a temporal stability of phase and amplitude response using electronic circuits, especially the active ones, demands tackling the temperature issue. The best approach usually makes use of a thermostatic system internal to the case, to maintain the temperature (as constant as necessary) [31]. This approach was not used in our case because one of the design requirements was to tightly limit the costs and the power consumption. We attempted to provide an accurate thermal insulation to reduce the temperature variations, without any active control. The design does not include the achievement of a high thermal stability able to provide a fine radiometric calibration as the case of transponders, which demand very high stability (lower than 0.1 °C) [32]. The approach followed here was to study the relationship between the phase response and the air temperature and possibly correct the data using a correction curve, to achieve a phase stability equivalent to deformation of the order of 2–3 mm.

**Figure 5:**
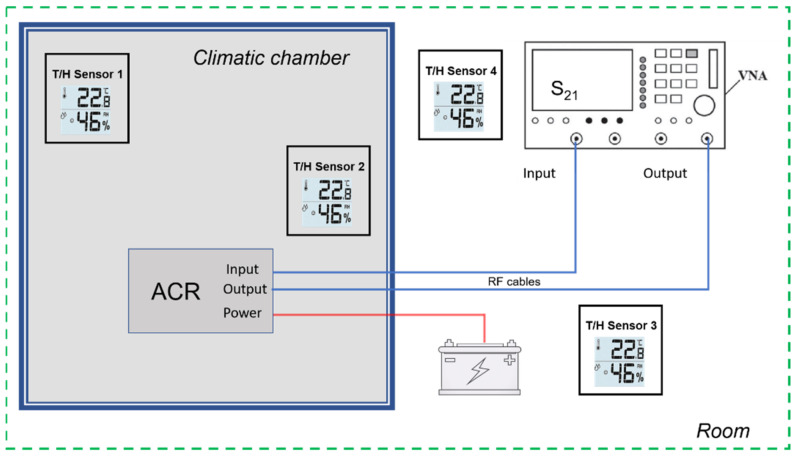
Measurement set-up for the RF chain test. Figures are dummy values to explain the setup.

To evaluate the reproducibility and trend of the thermal effect, we carried out different tests. We first measured the complex transfer function of the AR with different air temperatures, to estimate the stability of the amplifying section response. The AR was positioned inside a thermostatic chamber and air temperature was varied at single steps from 16° to 25°C for 6 h. This procedure guarantees operating in a thermal equilibrium state, moderately reproducing the operating conditions in the field. In Figure 5, the setup used for this experiment is depicted.

#### 3.1.2. The Results

**Figure 6:**
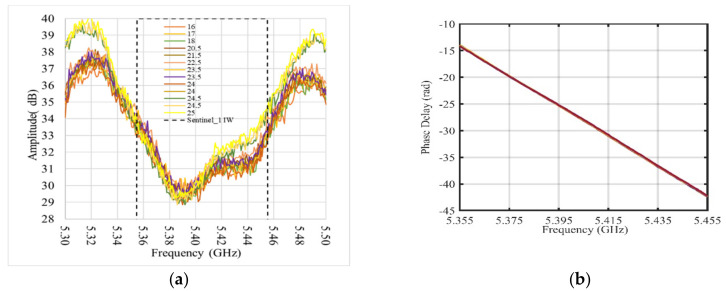
(**a**) Amplitude response in dB of the AR vs. frequency; dashed line marks the Sentinel-1 band; (**b**) phase delay introduced by AR vs. frequency. Different colors refer to temperature in degrees Celsius.

**Figure 7:**
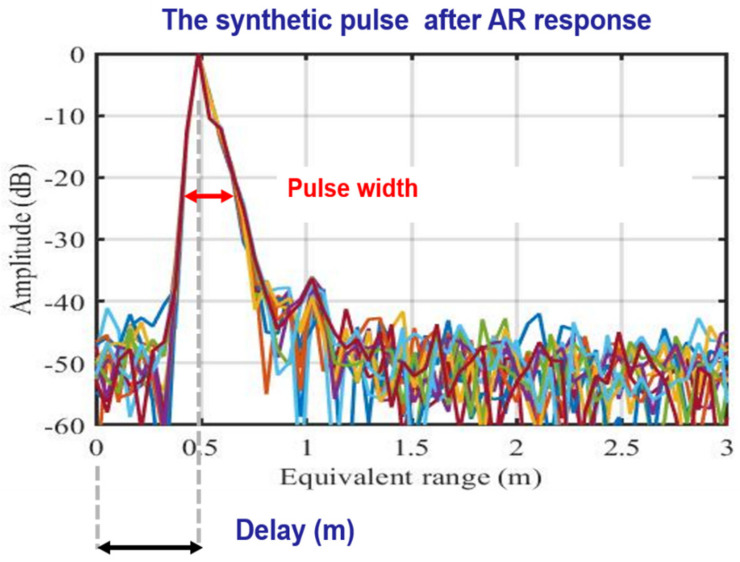
Synthetic pulse calculated using frequency response of the AR within the Sentinel-1 band. Different colors refer to temperature in degrees Celsius (see Figure 6). Pulse width and delay introduced by the AR are also indicated.

The results, amplitude, and phase of the S_21_ parameter measured by a VNA at different temperatures are shown in Figure 6a,b, respectively. In Figure 6a, the Sentinel-1 operating bandwidth is also marked (dashed line). The phase data shown in Figure 6b have been unwrapped. Observing these figures, although the amplitude shows a non-linear behavior, due to the band-pass filter response, the device appears fairly stable with temperature, and its phase response is linear. To evaluate the effect of this performance on the response of a synthetic pulse using the frequency data corresponding to the Sentinel-1 band, and to analyze the expected shape, data have been inverse fast Fourier transformed (IFFT) after a Hanning function windowing. The synthetic pulse response of the AR is shown in Figure 7, where the x-axis was converted in range units to allow estimating the delay introduced by the RF section, 0.5 m. The obtained value and the pulse shape guarantee that the shift does not significantly affect the pixel location. A detailed check of the pulse response of the AR, including the antennas and the RF connections, are described in the next section, dedicated to field experiment.

From the operational point of view, considering that the main goal of this device is to assist the use of InSAR for deformation monitoring, we tried to estimate the stability of the AR in terms of virtual displacement, i.e., the error introduced by the system in the retrieval of deformation. In SAR interferometry, the differential phase is usually estimated with respect to the wavelength, corresponding to the central frequency of the operated bandwidth, but the phase response, due to the synthetic nature of the pulse, is affected by the amplitude and phase response of the AR over the entire bandwidth. To estimate the effect of possible instabilities, we analyzed the trend of a statistical parameter, D (T (°C)), a virtual displacement, defined through the following Equation (2):

(2) $$D\left(f\left(\mathrm{GHz}\right),t\left({}^{\circ}C\right)\right)=\frac{1}{Nf}\overset{Nf}{\underset{1}{\sum }}\varphi_{i}(t\left({}^{\circ}C\right))\;\frac{c}{f_{i}4\pi }$$

*D* is obtained using the basic formula of radar interferometry to transform the phase to a displacement, stated by the following Equation (3):

(3) $$D\left[m\right]=\Delta \varphi \left[\mathrm{rad}\right]\;\frac{\lambda \;[m]}{4\pi }$$

where λ is the wavelength. The same factor, *c*/(4π *f*_i_), is present in Equation (3). 

*D* is calculated at temperature *t_i_*, averaging the phase, *φ*_i_, at each frequency, *f_i_*, over the entire number of frequencies, *N_f_*, number of measured frequencies belonging to the swept bandwidth, *N*_f_ ∗ (*f_i+1_* − *f_i_*); each phase is normalized to *f*_i_; *c* is the light velocity. This average can represent an estimate of the overall response of the AR to the incoming signal and it allows estimating the stability of the phase retrieved from interferometric processing as a displacement. 

**Figure 8:**
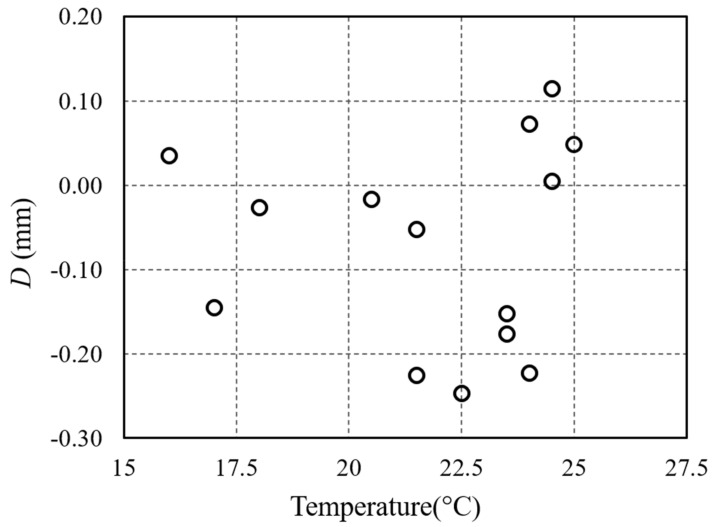
Parameter calculated by Equation (2) as a function of the temperature inside the thermostatic camera.

The variation of *D* versus the temperature measured inside the thermostatic camera is showed in Figure 8. The maximum variation of *D* obtained from the laboratory data is <0.4 mm, for a 15 °C to 25 °C temperature range, the standard deviation is 0.12 mm and the mean value −0.07 mm. Real conditions can usually cover a wider range of temperatures, and these values must be considered preliminary to the following tests, which were carried out with the system in its complete configuration. 

### 3.2. The Test in Controlled Environment at Short Range

**Figure 9:**
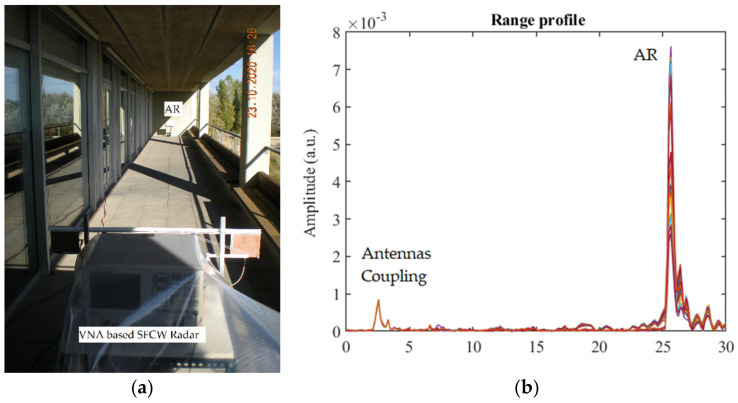
(**a**)Terrace setup. (**b**) Range profiles corresponding to the measurements (23–27 October 2020). Different colors refer to different acquisitions in time.

A second test of the AR was carried out, arranging a setup to measure the thermal stability of the AR at a short range, and also, in this case, using (as an input signal) an FMCW signal generated by the VNA. The test is based on the air temperature variations, measured through a sensor installed inside the AR. The location is an open-air terrace, which, although does not represent an ideal radar environment, due to the presence of walls and multiple reflections, allowed us to study the AR response, which was, however, clearly detected. This test allowed achieving the goal to characterize the effect of temperature variations on the AR response compatibly with the logistics issues of the environment. The VNA, as introduced in the previous section, is used as step frequency radar, simulating the Sentinel-1 signal. The AR is installed close to the wall, delimiting the bottom of the terrace, about 25 m far from the radar. Figure 9a shows a picture of the setup. We carried out three acquisitions in two very similar geometries; between the first and the second set of acquisitions—the radar and the AR were not moved, while in the third—the AR was moved a bit closer to the radar. In Table 2, we report details about the duration, number of acquisitions, temperature range of the radar acquisitions, and the parameters used to generate the testing signal. We summarize the analysis of the results of the last set of acquisitions (Table 2, case 3). In this case, the parameters to generate the signal were selected to obtain a larger unambiguous range. This value, 240 m, allows reducing potential aliasing artefacts in the profile caused by multiple reflections. Figure 9b shows the range profiles corresponding to the entire temporal slot of measurements, obtained at the different temperatures. The AR is well identified, together with the antenna coupling, and an instable target, which corresponds to an accessible door located along the aisle. 

**Figure 10:**
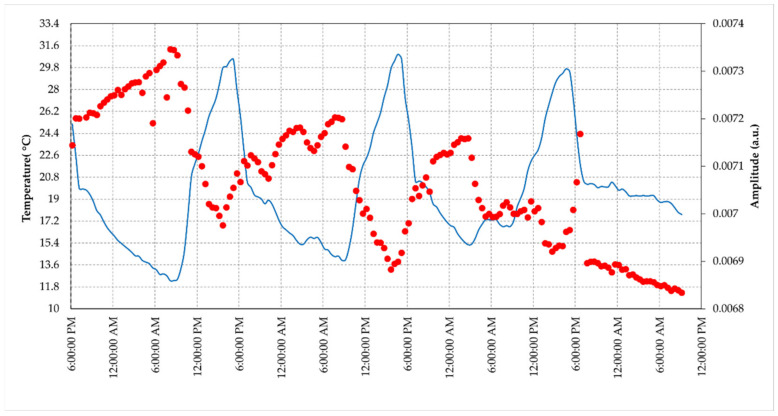
Amplitude response and temperature of the AR measured during the test at the terrace (30 October–3 November, 2020). The blue line represents temperature (left *y*-axis), while the red dots indicate the amplitude values (right *y*-axis).

Here, we refer to the phase measured by the radar in correspondence to the peak associated to the AR position, which represents the phase response of the AR, taking the first acquisition as reference, transformed to displacement, according to Equation (3). The temperature of the AR in this setup is measured every 15 min through a humidity and temperature sensor mounted inside the case. In Figure 10, we show the amplitude measured at the pixel, which identifies the AR as a function of the time; the slots of data were selected, avoiding data corrupted by external human disturbances unrelated to the AR response (e.g., people accidentally crossing the area). The amplitude fluctuations are confined within less than ±0.5 dB. It is worth noting that there is some correlation with the temperature. The basic statistical parameters calculated for this time lapse are resumed in Table 3.

**Figure 11:**
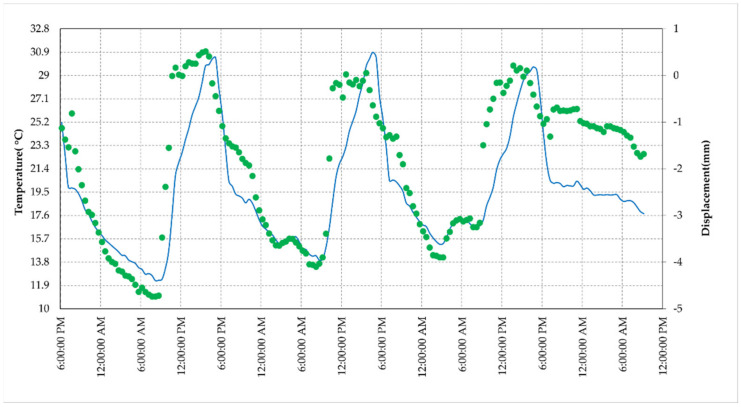
Displacement and temperature of the AR measured during the test at the terrace (30 October–3 November 2020). The blue line represents temperature (left *y*-axis) while the green dots indicate the displacements values (right *y*-axis).

As far as the phase trend is concerned, the retrieved displacements and the corresponding temperatures are plotted in Figure 11; Table 4 reports the statistical parameters of the dataset.

**Figure 12:**
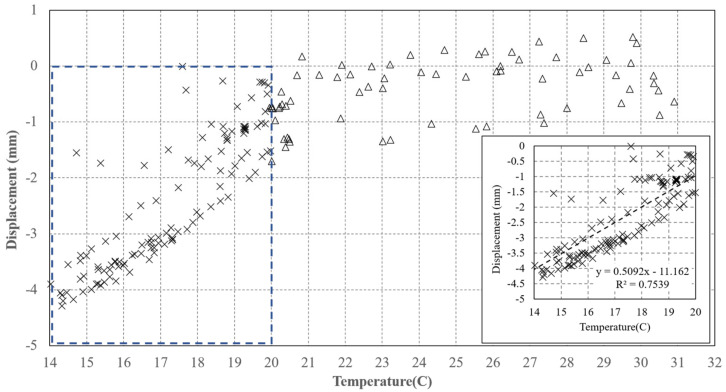
Virtual displacement of the AR versus temperature measured during the test at the terrace (30 October–3 November, 2020). Dashed rectangle magnified in the bottom left of the plot.

These results confirm that the amplitude and phase fluctuations are correlated to the temperature trend, as expected, when considering the typical thermal behavior of active components of an RF chain. To discover the type of relationship, we plot the phase data as a function of the temperature, shown in Figure 12. 

This graph clearly shows that in the “low temperature” range there is a linear trend, well fitted with a rate = 0.51 mm/°C line, and a bias of 11 mm. For temperatures above 20 °C, the variations are of a minor entity and are lower than 2 mm. Separating the data set into two sets, we define a discontinuous function, describing the dependence between the displacement and temperature, represented by Equation (4):

Displ = Displmeas—m *T − p   T < 20 °C Displ = Displmeas—Offset   T > 20 °C (4)

where: m = 0.51 (mm/°C), p = −11 (mm), Offset = −2 (mm).

It is worth noting that a very similar fit has also been obtained with the data acquired in the two previous data acquisitions.

## 4. The Field Test in Operative Conditions

Here we show the results of a field test, where the AR response is analyzed in a real condition, processing Sentinel-1 images; its phase stability is evaluated through a standard interferometric processing, which covers almost a one-year slot. In order to estimate the performances of the AR in a real situation, a prototype of the system and a PCR, rectangular side trihedral with a 0.65 m size, were installed on 22 October, 2019, in an agricultural field close to CTTC. Both reflectors were oriented to receive the Sentinel-1A and -1B signals in the descending orbit geometry. The AR (Latitude: 41.276895, Longitude: 1.986798) and the PCR (Latitude: 41.277137, Longitude: 1.986031) are still operating in January 2021. Considering that the use of the proposed AR focuses on InSAR applications, the main aim was to compare its phase response with respect to stable points as the PCR and some buildings close to it, representing an optimum phase stable reference. An analysis of the amplitude response is first performed considering that the phase accuracy is also influenced by the SCR of the AR.

### 4.1. Amplitude

A quantitative estimate of the RCS along the satellite line of sight LOS was carried out using field measurements, comparing the AR and PCR amplitude response in the acquired SAR images, and assuming that the RCS of the installed PCR corresponds to the expected maximum theoretical value, calculated according to the standard formula for rectangular side trihedral. The assessment of an absolute estimate of the RCS is a task planned for the next steps of the study, during the implementation of the final prototype. The conditions required to perform an accurate radiometric calibration were not guaranteed in the current installation due to the logistics and external factors. We were not able to reduce and evaluate the effect of multiple reflections with unstable characteristics of the agricultural soil, the supporting structure, and the influences of close targets [33]. 

**Figure 13:**
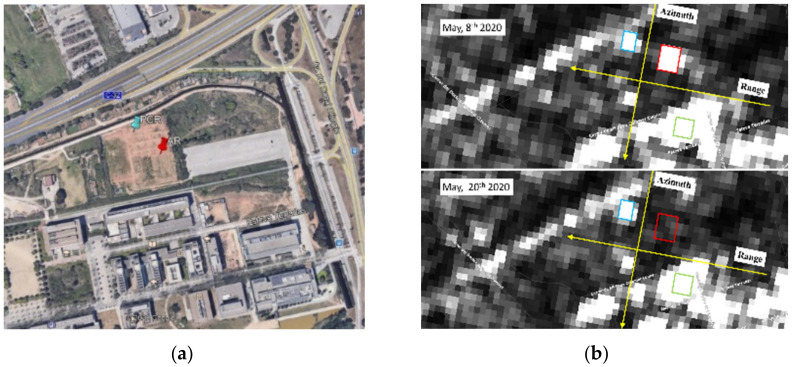
(**a**) Google map view of the area where the reflectors are installed. (**b**) Sentinel-1B, VV linear gamma zero orthorectified images, acquired on: 8 May 2020 (passive corner reflectors (PCR) and AR on), and 20 May 2020 (PCR on and AR off). Red and sky rectangles mark PCR and AR, respectively; green rectangle is the buildings area taken as reference. Images downloaded from [29].

Figure 13a shows a Google Maps^®^ view of the area, where both reflectors are installed. For a quick and easy check, the clear visibility of the AR and the PCR can also be verified by the readers in the SAR images, corresponding to the area where the AR is installed, using the Sentinel Hub browser provided by Synergy for ESA [27]. Figure 13b shows a zoom of two amplitude images of Sentinel-1, VV pol, with the indicated pixels corresponding to the PCR and AR strong responses: the bright pixels. Two different dates are shown: one when both AR and PCR were operating (8 May 2020), and the second when the AR was intentionally switched off (20 May 2020).

**Figure 14:**
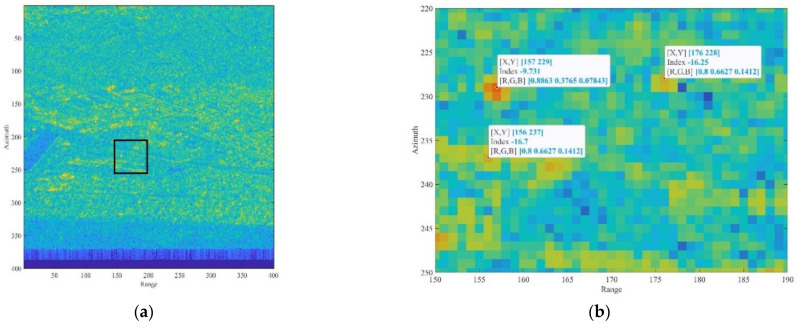
(**a**) Sentinel-1 amplitude image in radar coordinates with a black rectangle to localize the area where the AR and PCR are located. (**b**) Detail of the area with the indicated location and the amplitude (dB) value of the pixel corresponding to the AR in [157 229] (−9.7 dB), the PCR [157 229] (−16.3 dB). A point used as a reference for the interferometric processing is also marked [156 237].

**Figure 15:**
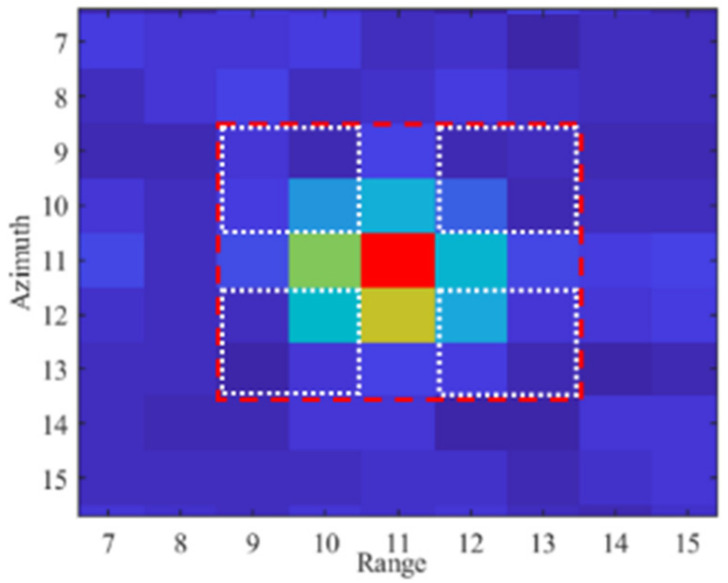
Zoomed view of the pixels surrounding the AR. The red dashed polygon indicates the pixels used to estimate the SCR. The four white rectangles encompass the clutter area, while the target is associated to the complementary remaining pixels distributed in cross shape.

To perform a quantitative analysis, we process different SLC images and use as reference the PCR amplitude response; the RCS of the AR (RCS_AR_) was estimated considering the responses of the two targets from the amplitude image, shown as Figure 14b and the theoretical value of the PCR (RCS_PCR_ = 33.4 dBm^2^). Calculating Δ_AP_ (dB) = intensity(AR) − intensity(PCR), the estimated value is RCS_AR_ = RCS_PCR_ + Δ_AP_ (dB) = (33.4 + 6.5) dB = 39.9 dBm^2^. This value is close to the one expected from the design and the laboratory test. The same image was also used in the following. Although in our study we focused on a data-based approach, i.e., evaluating the phase error from the final interferometric phase obtained after processing a temporal series of images, we also estimated the SCR directly form an amplitude image. The SCR was obtained by estimating the signal strength, averaging on 9 pixels distributed in a cross shape, and the clutter signal averaging on the remaining 16 pixels internal to the 5 × 5 square. Figure 15 depicts a graphic description of the pixel selection and Equation (5) the algebraic formula.

(5) $$I_{\mathrm{AR}}=I_{\mathrm{pixAR}}-\left(\frac{N_{\mathrm{CL}}}{N_{\mathrm{AR}}}\right)*I_{\mathrm{pixCl}}$$

In Equation (5), I_pixAR_ is the intensity, in dB, measured in the area associated to the targets, I_pixCL_, the intensity measured in the area associated to the clutter, and N_AR_ and N_CL_, the number of pixels used to calculate the average corresponding to the target and the clutter, respectively. The calculated SCR is 11 dB, corresponding to a theoretical accuracy lower than 1 mm [4], which fits with the performance required for the AR. 

### 4.2. Phase

**Figure 16:**
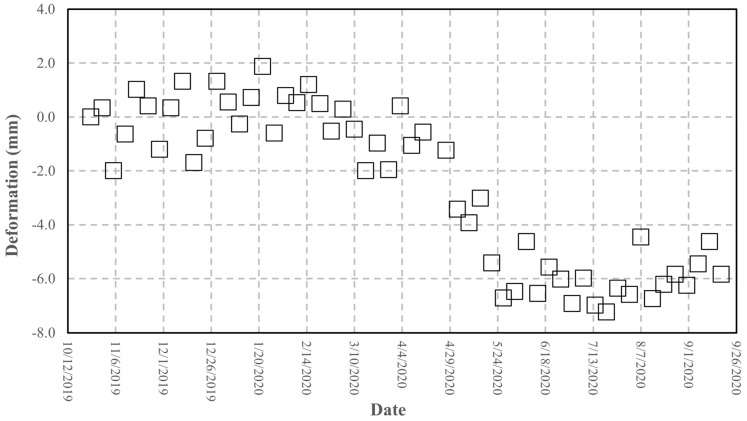
Cumulated deformation measured at the AR pixel retrieved from the Sentinel-1 image processing vs. the acquisition date, from 24 October 2019 to 18 September 2020.

**Figure 17:**
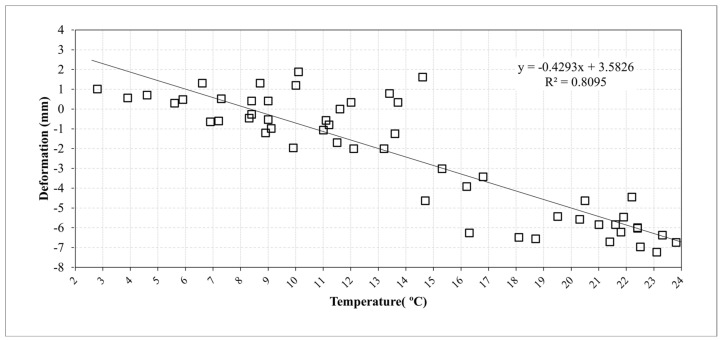
Cumulated deformation retrieved at the AR pixel obtained from the Sentinel-1 image processing vs. the air temperature, from 24 October 2019 to 18 September 2020 and fit line.

The interferometric processing applied to retrieve the differential phase uses, as reference, the phase response of some pixels corresponding to stable buildings, located tens of meters away from the AR to reduce the atmospheric effect (as lower as possible). In Figure 14b, one of these pixels is indicated. To estimate the AR phase stability, using Equation (3), we plotted the cumulated displacement versus the date of acquisition in Figure 16. A set of 55 Sentinel-1 images, SLC IW mode, descending orbit (orbit 37), and VV pol, covering the period from 24 October 2019 to 18 September 2020. Observing Figure 16, we note two stable periods separated by approximately 6 mm with a gradual passage. The same displacement data are plotted in Figure 17 vs. the air temperature available from a meteorological station, located a few kilometers far from the test site, and measured at 6:00 am GMT, a few minutes before the satellite passes. The range of air temperatures during the analyzed period is not very wide, about 20 °C, due to the climate of the site and the time of the passage.

**Figure 18:**
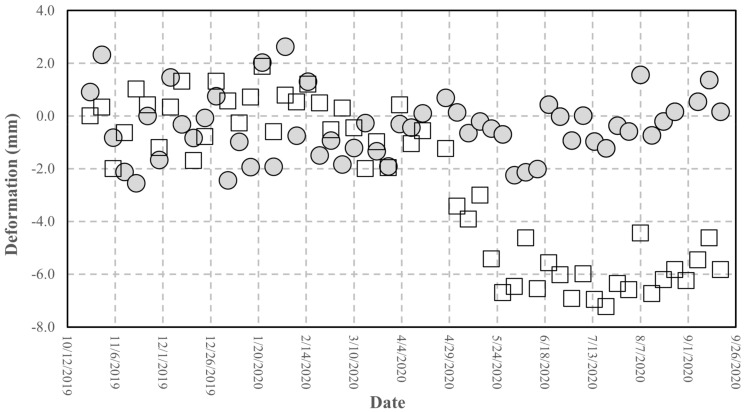
Cumulated deformation shown in Figure 16 (squares) corrected using Equation (4) (circles).

The trend of the phase vs. temperature, as observed in the previous experiments, highlight a linear effect of the temperature on the phase stability. This suggests applying a correction to improve the accuracy of the retrieved phase, using the formula already experienced in the short-range experiments. Figure 18 shows the comparison between the original data and the data corrected using Equation (4). A significant improvement is achieved: the statistical parameters reported in Table 5 show that the standard deviation drops to 1.6 mm with a bias of −2 mm. Although this result does not fully comply with the requirement of a millimetric accuracy, it represents a valuable starting point for planned improvements to the device presented, and also for applications where an accuracy of a few millimeters can be satisfactory.

## 5. Discussion

The device was tested at different levels and the results of the tests allow to outline the following points. The results obtained through the laboratory experiments carried out in a controlled environment are satisfactory. First, the RF section fits the design requirements in terms of pulse response and RCS. The measurement of the RCS, although obtained through an empirical method, comparing the AR pixel intensity to that of a PCR obtained from the SAR image (see Figure 13 and Figure 14), gives a value of 40 dBm^2^, which fits the design requirements. The stability of the amplitude response shows a variation lower than 2% in the short-range test and can also be considered satisfactory (see Figure 10. Amplitude response and temperature of the AR measured during the test at the terrace (30 October–3 November, 2020). The blue line represents temperature (left *y*-axis), while the red dots indicate the amplitude values (right *y*-axis)). It is worth noting that, during the design phase, the choice of the active components was not made through an exhaustive market survey, limiting to the achievement of satisfactory performances of the implemented RF chain. Thus, we cannot presume that a better performance could be obtained with different components, but this task is out of the main goal of this study. The RF gain, and the specifically-designed and implemented antennas, allow achieving a low current consumption, and low-cost, and the transfer function of the device assures a response with a negligible delay and distortion (see Figure 7). As far as the test of the device in a real case, as supported by other findings in literature [34,35], the influence of the temperature of the AR has been identified as the main factor inducing phase instability. Experimental data obtained with short-range experiments allowed to find a repeatable relationship between phase fluctuations and temperature. In the field test, consisting of retrieving the phase stability over a one-year lapse, and using the data of a series of Sentinel-1 images, the role of the temperature is confirmed. The importance of taking into account the temperature effect is evidenced by observing Figure 17, where a regression line fitted on the retrieved phase data show a high correlation between phase drift and temperature, with a correlation coefficient of 0.80. Applying Equation (4) to raw data resulted in a final uncertainty, after the whole Sentinel-1 interferometric processing, of a few millimeters. 

However, the study showed a main weak point, which can limit the performances of the implemented AR. The lack of an active temperature control system, which is responsible for the instability of its phase response. Such an approach falls outside the main rationale of this project because the introduction of a thermal control unit will increase the complexity of the device, affecting the energy consumption, and cost. The proposed approach, consisting of correcting the data offline, for some long-term applications where data are not processed in real time, does not represent a critical issue. To moderate the effect of the thermal instability, one of the planned activities in the next version of the AR under development is to reduce the size of the RF section using a specifically-designed single board for the amplifying section. This more compact solution could improve the RF performance and reduce cost. At the same time, the mitigation of the influence of the external temperature for a small, single, component, could be achieved with an improved thermal insulation and with negligible increase of the power consumption.

## 6. Conclusions

This paper described the design, implementation, and test of a low-cost, moderate performance active reflector to be used with Sentinel-1 SAR. The tests were carried out in a laboratory, in a controlled environment, and in a real case, in the field, comparing its behavior to stable targets and using a consolidated InSAR processing chain. The device demonstrated satisfactory performances in terms of RCS, guaranteeing high visibility in an agricultural field, providing adequate SNC. The phase behavior, compared to that of stable points, retrieved by processing a one-year Sentinel-1 image set, enhanced a non-negligible instability, due to the external temperature variations. An empirical approach, consisting of making use of a calibration curve, obtained experimentally, allows achieving a phase uncertainty closer to that of ARs presently available on the market, which claim a 1 mm capability. However, most of the tests available in literature are carried out for temporal intervals (shorter than what was discussed here—eleven months). The results of the several tests analyzed in this study show that the implemented prototype, using a simple correction formula drawn on the bases of experimental data, can improve the performance of the sensor to finally provide a ±2 mm uncertainty, a value that can be considered satisfactory in situation where the installation of conventional PCR or high-cost AR is not advisable. Finally, concerning a rough estimate of the overall cost, in the current version, it is below 1000 euros, but the planned integration of the RF section could reduce to a few hundred euros, the price of commercial development, collocating the device as a low-cost apparatus with fair performance—one that could operate in remote areas with an acceptable risk of losing the device.

## Figures and Tables

**Table 1:** Main AR performances.

Operating mode	Single: ascending or descending
Polarization	One single linear co- or cross-polarization
Radar Cross Section (RCS)	40 dBm^2^
Antenna gain	17 dB
RF gain	42 dB
Power consumption	<0.05 A @ 12V

**Table 2:** Main data of the terrace experiment configurations; Runamb: stand for unambiguous range.

Case	Acquisition Start (Date, Time)	Acquisition Stop (Date, Time)	Number of Acquisitions	Temp Range (°C)	Sampling Time Number of Frequencies
1	23.10.20 16:10:14	27.10.20 14:11:27	189	15–36	30 min/Nf = 201 (Runamb = 30 m)
2	27.10.20 14:41:28	29.10.20 17:12:09	101	14–31.5	30 min/Nf = 201 (Runamb = 30 m)
3	29.10.20 17:42:10	03.11.20 21:17:06	199	12.5–31	30 min/Nf = 1601 (Runamb = 240 m)

**Table 3:** Amplitude statistical parameters calculated for the terrace experiment data set.

Mean	SD	SD/Mean (%)	Max	Min
0.00706	0.00014	2	0.00734	0.00683

**Table 4:** Main data of the terrace experiment configuration.

Mean (mm)	SD (mm)	Max (mm)	Min (mm)
−2.0	1.6	0.5	−5.4

**Table 5:** Main statistical parameters obtained from campus acquisitions.

	Mean (mm)	SD (mm)	Max (mm)	Min (mm)
Uncorrected data	−2.6	3	1.9	−7.2
Corrected data	−2.0	1.6	0.5	−5.4

## Data Availability

Data are available at CTTC.

## References

[1] Ferretti A., Prati C., Rocca F. (2001). Permanent scatterers in SAR interferometry. IEEE Trans. Geosci. Remote Sens..

[2] Crosetto M., Monserrat O., Cuevas-González M., Devanthéry N., Crippa B. (2016). Persistent scatterer interferometry: A review. ISPRS J. Photogramm. Remote Sens..

[3] Jauvin M., Yan Y., Trouvé E., Fruneau B., Gay M., Girard B. (2019). Integration of Corner Reflectors for the Monitoring of Mountain Glacier Areas with Sentinel-1 Time Series. Remote Sens..

[4] Garthwaite M. (2017). On the Design of Radar Corner Reflectors for Deformation Monitoring in Multi-Frequency InSAR. Remote Sens..

[5] Doerry A.W. (2008). Reflectors for SAR Performance Testing.

[6] Chengfan L., Jingyuan Y., Zhao J., Zhang G., Shan X. (2012). The selection of artificial corner reflectors based on RCS analysis. Acta Geophys..

[7] Dheenathayalan P., Caro Cuenca M., Hoogeboom P., Hanssen R. (2017). Small Reflectors for Ground Motion Monitoring with InSAR. IEEE Trans. Geosci. Remote Sens..

[8] Quin G., Loreaux P. (2013). Submillimeter Accuracy of Multipass Corner Reflector Monitoring by PS Technique. IEEE Trans. Geosci. Remote Sens..

[9] Parker A., Featherstone W., Penna N., Filmer M., Garthwaite M.C. (2017). Practical Considerations before Installing Ground-Based Geodetic Infrastructure for Integrated InSAR and cGNSS Monitoring of Vertical Land Motion. Sensors.

[10] Crosetto M., Luzi G., Monserrat O., Barra A., Cuevas-González M., Palamá R., Krishnakumar V., Wassie Y., Mirmazloumi S.M., Espín-López P. (2020). Deformation monitoring using SAR Interferometry and active and passive reflectors. Int. Arch. Photogramm. Remote Sens. Spat. Inf. Sci..

[11] Komac M., Holley R., Mahapatra P., van der Marel H., Bavec M. (2015). Coupling of GPS/GNSS and radar interferometric data for a 3D surface displacement monitoring of landslides. Landslides.

[12] Ferrer P.J., Lopez-Martinez C., Aguasca A., Pipia L., Gonzalez-Arbesu J.M., Fabregas X., Romeu J. (2011). Transpolarizing Trihedral Corner Reflector Characterization Using a GB-SAR System. IEEE Geosci. Remote Sens. Lett..

[13] Freeman A., Shen Y., Werner C.L. (1990). Polarimetric SAR calibration experiment using active radar calibrators. IEEE Trans. Geosci. Remote Sens..

[14] Sarabandi K., Oh Y., Ulaby F.T. (1992). Performance characterization of polarimetric active radar calibrators and a new single antenna design. IEEE Trans. Antennas Propag..

[15] Satake M., Fujita M., Hanado H., Horie H., Sato K., Ochiai S. Calibration experiments of ERS-1 SAR with active radar calibrator in Japan. Proceedings of the IGARSS ’94—1994 IEEE International Geoscience and Remote Sensing Symposium.

[16] Woode A., Desnos Y., Jackson H. (1992). The development and first results from the ESTEC ERS-1 active radar calibration unit. IEEE Trans. Geosci. Remote Sens..

[17] Hawkins R., Teany L., Srivastava S., Tam S. (1997). RADARSAT precision transponder. Adv. Space Res..

[18] Hounam D., Zwick H., Rabus B. A permanent response SAR transponder for monitoring ground targets and features. Proceedings of the 7th European Conference on Synthetic Aperture Radar.

[19] Hole J., Holley R., Giunta G., De Lorenzo G., Adam A.M. Insar Assessment of Pipeline Stability using Compact Active Transponders. Proceedings of the Fringe 2011 Workshop.

[20] Mahapatra P.S., Samiei-Esfahany S., van der Marel H., Hanssen R.F. (2014). On the use of transponders as coherent radar targets for SAR interferometry. IEEE Trans. Geosci. Remote Sens..

[21] Döring B., Schmidt K., Jirousek M., Rudolf D., Reimann J., Raab S., Antony J., Schwerdt M. (2013). Hierarchical bayesian data analysis in radiometric SAR system calibration: A case study on transponder calibration with RADARSAT-2 data. Remote Sens..

[22] Li L., Liu G., Hong J., Ming F., Wang Y. (2019). Design and Implementation of a Multi-Band Active Radar Calibrator for SAR. Remote Sens..

[23] Cabria L., García J.Á., Gutiérrez-Ríos J., Tazón A., Vassal’lo J. (2009). Active Reflectors: Possible Solutions Based on Reflect arrays and Fresnel Reflectors. J. Antennas Propag..

[24] METASENSING Radar Solucions. https://www.geomatics.metasensing.com/ecr-c.

[25] Brunfeldt D.R., Ulaby F.T. (1984). Active reflector for radar calibration. IEEE Trans. Geosci. Remote Sens..

[26] Luzi G., Fernandez E., Mirá-Perez F., Crosetto M. A Low Cost Active Corner Reflector to assist Snow Monitoring through Sentinel/1 images. Proceedings of the 14th European Conference on Antennas and Propagation (EuCAP2020).

[27] EO Browser. https://apps.sentinel-hub.com/eo-browser/.

[28] Devanthéry N., Crosetto M., Monserrat O., Cuevas-González M., Crippa B. (2014). An Approach to Persistent Scatterer Interferometry. Remote Sens..

[29] Copernicus https://scihub.copernicus.eu/dhus/#/home.

[30] METEOCAT Catalan Meteorological service https://www.meteo.cat.

[31] Raab S., Doering B.J., Rudolf D., Reimann J., Schwerdt M. Analysis of an Improved Temperature Management Concept for SAR System Calibration Transponders. Proceedings of the EUSAR 2016: 11th European Conference on Synthetic Aperture Radar.

[32] Rudolf D., Raab S., Döring B.J., Jirousek M., Reimann J., Schwerdt M. Absolute Radiometric Calibration of the Novel DLR “Kalibri” Transponder. Proceedings of the 2015 German Microwave Conference.

[33] Doerry A.W., Brock B.C. (2009). Radar Cross Section of Triangular Trihedral Reflector with Extended Bottom Plate 2009.

[34] Jirousek M., Döring B., Rudolf D., Raab S., Schwerdt M. Development of the highly accurate DLR Kalibri Transponder. Proceedings of the EUSAR 2014—10th European Conference on Synthetic Aperture Radar.

[35] Rabb S. (2016). Development and Implementation of an Efficient Temperature Management System for SAR System Calibration Transponders. Master’s Thesis.

